# Estradiol improves cardiovascular function through up-regulation of SOD2 on vascular wall

**DOI:** 10.1016/j.redox.2014.11.001

**Published:** 2014-11-15

**Authors:** Zhaoyu Liu, Yulan Gou, Hongyu Zhang, Houjuan Zuo, Haimou Zhang, Zhengxiang Liu, Dachun Yao

**Affiliations:** a Internal Medicine of Tongji Hospital, Tongji Medical College, Huazhong University of Science and Technology, Wuhan 430030, PR China; b Department of Neurology, Wuhan No. 1 Hospital, #215 Zhongshan Road, Wuhan 430022, PR China; c Department of Hematology, Peking University ShenZhen Hospital, ShenZhen 518036, PR China; d School of Life Sciences, Hubei University, Wuhan 430062, PR China

**Keywords:** Endothelium, Estrogen receptor, Gene therapy, Mitochondrial function, Reactive oxygen species

## Abstract

Epidemiological studies have shown that estrogens have protective effects in cardiovascular diseases, even though the results from human clinical trials remain controversial, while most of the animal experiments confirmed this effect, but the detailed mechanism remains unclear. In this study, we found that estradiol (E2) treatment significantly increases the expression of mitochondrial superoxide dismutase (SOD2) in mice and in vitro in human aorta endothelial cells. Further investigation shows that E2 up-regulates SOD2 through tethering of estrogen receptor (ER) to Sp1 and the increased binding of Sp1 to GC-box on the SOD2 promoter, where ERα responses E2-mediated gene activation, and ERβ maintains basal gene expression level. The E2/ER-mediated SOD2 up-regulation results in minimized ROS generation, which highly favors healthy cardiovascular function. Gene therapy through lentivirus-carried endothelium-specific delivery to the vascular wall in high-fat diet (HFT) mice shows that the SOD2 expression in endothelial cells normalizes E2 deficiency-induced ROS generation with ameliorated mitochondrial dysfunction and vascular damage, while SOD2 knockdown worsens the problem despite the presence of E2, indicating that E2-induced SOD2 expression plays an important vasculoprotective role. To our knowledge, this is the first report for the mechanism by which E2 improves cardiovascular function through up-regulation of SOD2 in endothelial cells. In turn, this suggests a novel gene therapy through lentivirus-carried gene delivery to vascular wall for E2 deficiency-induced cardiovascular damage in postmenopausal women.

## Introduction

Many epidemiological studies have shown that postmenopausal women who use estrogens are at lower risk for cardiovascular disease (CVD) than those who do not use estrogens, suggesting a beneficial action of endogenous estrogen [1–3]. The vasculoprotective effect of estrogen has been demonstrated clearly in many animal models to prevent atherosclerosis development, such as in apolipoprotein E-deficient (ApoE^−/−^), low density lipoprotein receptor-deficient (LDLr^−/−^) mice [4–6] and high-fat diet (HFD) mice [7]. However, the large randomized controlled clinical trials did not confirm the preventive action of estrogens against coronary heart disease (CHD) [8]. This discrepancy has been attributed to the importance of age in the action of estrogens on endothelial function [9], the differences of endogenous estrogen and conjugated equine estrogens (CEEs) and the differences between oral and transdermal drug delivery [10].

Estrogens, primary examples of female sex steroids, control cellular growth, differentiation, female reproductive functions, and cardiovascular systems [11]. Estradiol (E2), the main form of estrogen, interacts with two estrogen receptors (ER), ERα and ERβ [12], which exert their effects through diverse signaling pathways that mediate the genomic and nongenomic events [13,14]. Membrane-initiated effects regulate ion channels and signal transduction pathways through post-translation modification or protein–protein interaction [15,16], while genomic effects regulate gene transcription either through the direct binding of ER dimers to estrogen response elements (ERE) on related gene promoters [17] or by cross-talk or tethering to other transcription factors, such as activation protein-1 (AP1) [18], NFκB [19] or specificity protein-1 (Sp1) [20].

Excessive reactive oxygen species (ROS) generation plays an important role in a number of cardiovascular diseases, including hypertension, atherosclerosis and myocardial ischemia/reperfusion (I/R) injury [21]. The antioxidant enzyme SOD2 (mitochondrial superoxide dismutase) catalyzes dismutation of mitochondrial O_2_^•−^, it has been reported to have a protective effect on vascular function. SOD2 deficiency results in increased ROS generation with subsequent mitochondrial dysfunction and oxidative stress [22,23]. SOD2 was used for the gene therapy in the animal model of CVD, and the results showed that SOD2 gene transfer reverses vascular dysfunction only in the absence of atherosclerosis plaque [21,24], this may indicate that the endothelial cells play a dominant role in vasculoprotective action as long as the endothelial cells irreversibly lose the function, the SOD2 therapy will not work, this is consistent with the recent findings [4].

In an effort to identify the molecular mechanism for the E2-mediated vasculoprotective effect, we have found that the SOD2 expression is significantly increased in endothelial cells under the E2 treatment. Further investigation shows that E2 up-regulates SOD2 through tethering of ER to Sp1 and the increased binding to GC-box on the SOD2 promoter, where both ERα and ERβ play an essential role in this kind of regulation. SOD2 up-regulation results in minimized ROS generation, which largely favors cardiovascular function. Gene therapy through lentivirus-carried endothelium-specific delivery to vascular wall in high-fat diet (HFD) mice shows that the SOD2 expression normalizes E2 deficiency-induced ROS generation with ameliorated cardiovascular damage. We conclude that E2 improves cardiovascular function through up-regulation of SOD2 expression on vascular wall.

## Experimental procedure

### Materials and methods

Primary human aorta endothelial cells (HAECs, obtained from Lonza) were conditionally immortalized by hTERT expression with extended life span [25,26]. Cells were maintained in EBM-2 medium (from Lonza) with all the supplements. The HAECs were treated with ethanol (vehicle), 100 nM E2 (from Sigma), or E2 plus 10 µM ICI 182,780 (ICI, from Tocris Bioscience) for 48 h, then the cells were harvested for further analysis. The antibodies for β-actin (sc-47778), 3-nitrotyrosine (3-NT, sc-55256), SOD2 (sc-30080), Sp1 (sc-59) and Sp3 (sc-644) were obtained from Santa Cruz Biotechnology, and the antibodies for ERα (ab37438) and ERβ (sc-137381) were purchased from Abcam. Nuclear extracts were prepared using the NE-PER Nuclear and Cytoplasmic Extraction Reagents Kit (Pierce Biotechnology). Protein concentration was measured by Coomassie Protein Assay Kit (Pierce Biotechnology) using BSA as a standard. Plasmid DNA was transfected by Lipofectamine™ reagent (Invitrogen). siRNA against Sp1, Sp3, ERα and ERβ or non-specific siRNA (from Ambion) were transfected using Oligofectamine reagent (Invitrogen) according to the manufacturers' instructions.

### Construction of plasmids and vectors

Human genomic DNA was prepared from HAECs. The SOD2 gene promoter (2 kb upstream of the transcription start site plus first exon) was amplified by PCR and subcloned into pGL3-basic vector. To localize estrogen response element (ERE) on the SOD2 promoter, the related deletion constructs were generated by PCR methods and the indicated ERE or GC box mutation plasmids were generated using the Site-directed Mutagenesis Kit from Promega. The cDNA for SOD2 (from Open Biosystems) was subcloned into pcDNA3.1 for generation of expression plasmids. All the vectors were verified by sequencing, and the detailed information is available upon request.

### RT reaction and real-time quantitative PCR

Total RNA from treated cells was extracted by RNeasy Mini Kit or RNeasy Micro Kit (Qiagen), and the RNA was reverse transcribed by Omniscript RT kit (Qiagen). Real-time quantitative PCR was run on iCycler iQ (Bio-Rad) with the Quantitect SYBR green PCR kit (Qiagen). PCR was performed by denaturing at 95 °C for 8 min, followed by 45 cycles of denaturation at 95 °C, annealing at 60 °C, and extension at 72 °C for 10 s, respectively. 1 µl of each cDNA was used to measure target genes. The results were normalized by β-actin.

### Luciferase reporter assay

1.0×10^5^ Cells were seeded in a 6-well plate with completed medium to grow until they reach 80% confluence. The related luciferase reporter plasmids (3 µg) and 0.2 µg pRL-CMV-Luc*Renilla* plasmid (from Promega) were transiently cotransfected. Some experiments need to cotransfect the siRNA oligonucleotides. After 12 h transfection, the cells were washed and treated for 48 h with either ethanol control, 100 nM E2 or E2 plus 10 µM ICI 182,780. The cells were harvested and the luciferase activity assays were carried out using the Dual-Luciferase™ Assay System (Promega), and transfection efficiencies were normalized using a cotransfected *Renilla* plasmid.

### Western blotting

Cells were lysed in ice-cold lysis buffer (0.137 M NaCl, 2 mM EDTA, 10% glycerol, 1% NP-40, 20 mM Tris base, pH 8.0) plus protease inhibitor cocktail (Sigma), and the proteins were separated in 10% SDS-PAGE and further transferred to PVDF membrane, the membrane was incubated with appropriate antibodies, washed and incubated with HRP-labeled secondary antibodies, then the blots were visualized by ECL+plus Western Blotting Detection System (Amersham), and the blots were quantitated by IMAGEQUANT, and the final results were normalized by β-actin.

### Chromatin immunoprecipitation (ChIP)

The procedure used here is described by Metivier et al. with minor modification [27]. Briefly, treated cells were washed and crosslinked using 1% formaldehyde for 20 min and terminated by 0.1 M glycine. Cell lysates were sonicated and centrifuged. 500 µg protein were pre-cleared by BSA/salmon sperm DNA plus preimmune IgG and a slurry of Protein A Agarose beads. Immunoprecipitations were performed with the indicated antibodies, BSA/salmon sperm DNA and a 50% slurry of Protein A agarose beads. Input and immunoprecipitates were washed and eluted, then incubated with 0.2 mg/ml Proteinase K for 2 h at 42 °C followed by 6 h at 65 °C to reverse the formaldehyde crosslinking. DNA fragments were recovered by phenol/chloroform extraction and ethanol precipitation. No bands were observed after immunoprecipitation with pre-immune IgG. A ~150 bp fragment from the SOD2 promoter was amplified by real-time PCR (qPCR).

### Measurement of ROS generation

Treated cells were seeded in a 96-well plate and incubated with 10 µM CM-H2DCFDA (Invitrogen) for 45 min at 37 °C, then the intracellular formation of reactive oxygen species (ROS) was measured at excitation/emission wavelengths of 485/530 nm using an FLx800 microplate fluorescence reader (Bio-Tek). The data were normalized as arbitrary units [28]. Levels of an oxidative marker 3-nitrotyrosine was measured by western blots.

### DNA affinity precipitation assay (DAPA)

Biotin-labeled sense and antisense oligonucleotides with fragment (−270 to −138) of human SOD2 promoter were synthesized, annealed and purified as the DAPA probe. 200 µg nuclear extracts in binding buffer (60 mM KCl, 12 mM HEPES, pH 7.9, 4 mM Tris–HCl, pH 7.5, 5% glycerol, 0.5 mM EDTA, 1 mM DTT and protease inhibitors) were pre-cleared by 3 µg of scrambled double-strand DNA supplemented with pre-equilibrated Tetralink™ Avidin Resin (Promega). The pre-cleared nuclear extracts were further incubated with 2 µg of DAPA probe at 4 °C for 2 h with gentle rotation, then 20 µl pre-equilibrated Tetralink™ Avidin Resin was added to incubate for another 1 h. Beads were pelleted and washed with buffer, then boiled for 5 min in SDS-PAGE gel loading buffer for western blotting analysis.

### Generation of Tie2-driven SOD2 expression lentivirus

The mouse genomic DNA was purified from C57BL/J6 wild type mouse, and the endothelium-specific Tie2 promoter (−2000 upstream plus exon 1) was amplified by PCR, fused with the mouse SOD2 cDNA (obtained from Open Biosystems), then they were subcloned into pLVX-Puro vector (from Clontech), and the Tie2-empty, or Tie2-SOD2 lentivirus, was expressed through Lenti-X™ Lentiviral Expression Systems (from Clontech) according to manufactures’ instructions. The virus was further purified, concentrated and titrated to reach ~2×10^8^ MOI/mL for the tail vein injection to infect the experimental mice.

### Generation of Tie2-driven SOD2 shRNA lentivirus

The shRNA for scramble or SOD2 were synthesized and fused with mouse Tie2 promoter (−2000 upstream plus exon 1), then subcloned into pLVX-shRNA2 vector (from Clontech), and the Tie2-scramble (CTL), or Tie2-shSOD2 lentivirus was expressed through Lenti-X™ shRNA Expression Systems (from Clontech) according to manufactures' instructions.

### In vivo mice experiments

The animal study protocol was reviewed and approved by the Institutional Animal Care and Use Committee of our institute. The female C57BL/6J mice were housed 4 or 5 per cage on a 12:12-h light-dark cycle and were given phytoestrogen-free commercial rodent chow and water ad libitum on arrival. At 4 weeks of age, the mice received either sham or bilateral ovariectomy (OVX) surgery with high-fat diet (HFD, 60% calories from fat, Research Diets Cat. #D12492) through the rest of the experiments. At 6 weeks, mice received treatments consisting of 90-day time release pellets (Cat #: NE-121, from Innovative Research of America) that were implanted subcutaneously via a ~3-mm incision on the dorsal aspect of the neck. Hormone pellets contained 0.72 mg of E2, while placebo pellets contained the same matrix as the E2 pellets but with no hormone [4,29]. The mice also received tail vein injection of 150 µl lentivirus (2×10^8^ MOI) for either Tie2-empty (CTL), Tie2-↑SOD2 or Tie2-shSOD2 continuously twice within a 2-day interval. At 19 weeks, the old estradiol release pellets were replaced by the new pellets to achieve continuous E2 treatment. At 32 weeks, the overnight-fasted mice were anesthetized, the body weight was measured, the tissues, including heart, aorta, liver, kidney and hypothalamus, were isolated for mRNA analysis, and the blood was collected for measuring plasma estrogen (E2) and lipids, including total cholesterol and triglyceride. The MECs were isolated from the heart and aorta and cultured in vitro for the further analysis of SOD2 activity and mitochondrial function. The aortas were isolated to measure in vivo superoxide release and vessel tension. In some treatments, the hearts or aortas were dissected and snap-frozen in OCT compound. 10 µm sections were cut by clean microtome and mounted on PEN-membrane slides (2.0 µm, Leica) for isolation of mouse endothelial cells (MECs) using Laser Capture Microdissection (LCM) for mRNA analysis. The details for those mice and their treatments are shown in Table 1.

### Plasma analysis for E2 and lipids

E2 level were analyzed on plasma from individual mice obtained at sacrifice using the E2 ELISA kit (Cat #: ADI-900-174 from Enzo). The total cholesterol (TC) and triglyceride (TG) in plasma were measured using a GM7 Micro.-Stat Rapid Multiassay Analyser (Analox) according to manufactures' instructions.

### In vivo superoxide release

The superoxide anion (O_2_^•−^) release from the aorta was determined by a luminol–EDTA–Fe enhanced chemiluminescence (CL) system supplemented with dimethyl sulphoxide–tetrabutyl-ammonium chloride (DMSO–TBAC) solution for extraction of released O_2_^•−^ from tissues as described previously [28]. The superoxide levels were calculated from the standard curve generated by the xanthine/xanthine oxidase reaction.

### Isolation of mouse endothelial cells (MECs)

Isolation of endothelial cells from the heart and aortas was performed following the previously described procedure [30]. The isolated endothelial cells were further characterized by immunofluorescence staining with an antibody to the von Willebrand factor (vWF). The passage P3–P5 was used for further in vitro analysis.

### Preparation of mitochondrial fraction

The mitochondrial fraction was prepared from the above isolated MECs using differential centrifugation [31] with minor modification. Briefly, treated cells were harvested and cell pellets were homogenized in the buffer containing 15 mM Tris buffer, pH 7.6; 0.25 M sucrose; 1 mM MgCl_2_; 1 mM EDTA; 1 mM dithiothreitol (DTT) and the mixture of protease inhibitor cocktail (Sigma). Cell homogenates were centrifuged at 800*g* at 4 °C for 8 min, subsequent supernatants were further centrifuged at 16,000*g* at 4 °C for 15 min. The pellets were resuspended in homogenization buffer and centrifuged at 16,000*g* at 4 °C for 20 min. The subsequent pellets were resuspended and collected as the mitochondrial fractions for the SOD2 activity assay.

### Evaluation of SOD2 activity

The SOD activity from the mitochondrial fraction of MECs was measured as described previously [32]. Briefly, a stable O_2_^•−^ source was generated through the conversion action of xanthine oxidase (XOD) from xanthine and was mixed with chemiluminescent (CL) reagents to achieve a stable light emission. The SOD2 sample injection can scavenge O_2_^•−^ and the subsequent decrease of chemiluminescent response is proportional to the SOD2 activity. This system can have a detection limit of 0.001 U/ml with the linear range of 0.03–2.00 U/ml. The results were normalized by protein concentration and were expressed as Units/mg proteins (U/mg).

### Measurement of mitochondrial function

The intracellular ATP level was determined by the luciferin/luciferase-induced bioluminescence system. An ATP standard curve was generated at concentrations of 10^−12^–10^−3^ M. Intracellular ATP levels were calculated and expressed as nmol/mg protein. The Mitochondrial Membrane Potential (MMP, Δ*Ψ*_m_) was measured by 3,3′-dihexiloxadicarbocyanine (DiOC_6_), cells were trypsinized, resuspended and incubated with 0.2 µM DiOC_6_ for 20 min at 37 °C, and cells were further treated with 1 µM propidium iodide for 30 min. The DiOC_6_ fluorescence was measured by FACS at an excitation/emission wavelength of 485/500 nm [28]. The caspase-3 activity was determined by the ApoAlert caspase assay kit (Clontech). Treated cells were harvested and 50 µg of proteins were incubated with the fluorogenic peptide substrate Ac-DEVD-7-amino-4-trifluoromethyl coumarin (AFC). The initial rate of free AFC release was measured using an FLx800 microplate reader (Bio-Tek) at excitation/emission wavelengths of 380/505 nm, and enzyme activity was calculated as pmol/min/mg [28].

### Monitoring of vascular function

The vascular function was evaluated by measurement of vessel tension. The vessel tension in mice aortas was measured in a vessel myograph system (from DMT). The aortas were isolated and immersed in Krebs bicarbonate buffer (118 mmol/l NaCl, 4.7 mmol/l KCl, 25 mmol/l NaHCO3, 1.2  mmol/l KH_2_PO_4_, 1.2 mmol/l MgSO_4_, 2.5 mmol/l CaCl_2_, and 5 mmol/l glucose) and then suspended by 2 tungsten wires mounted in vessel myograph system. To study vasodilator responses, the rings were preconstricted with phenylephrine, and the acetylcholine (Ach, 10^−10^–10^−4^ M) was injected at the plateau of the phenylephrine-induced contraction [33].

### Statistical analysis

The data were given as mean±standard deviation (S.D.), all experiments were performed at least in triplicate unless otherwise indicated. All analyses were performed using GraphPad Prism 6 statistical software. The student unpaired *t* test or ANOVA were used to determine statistical significance of different groups, a *P* value <0.05 was considered significant.

## Results

### Female mice have significant higher expression of SOD2 than male mice in the cardiovascular system

**Fig. 1:**
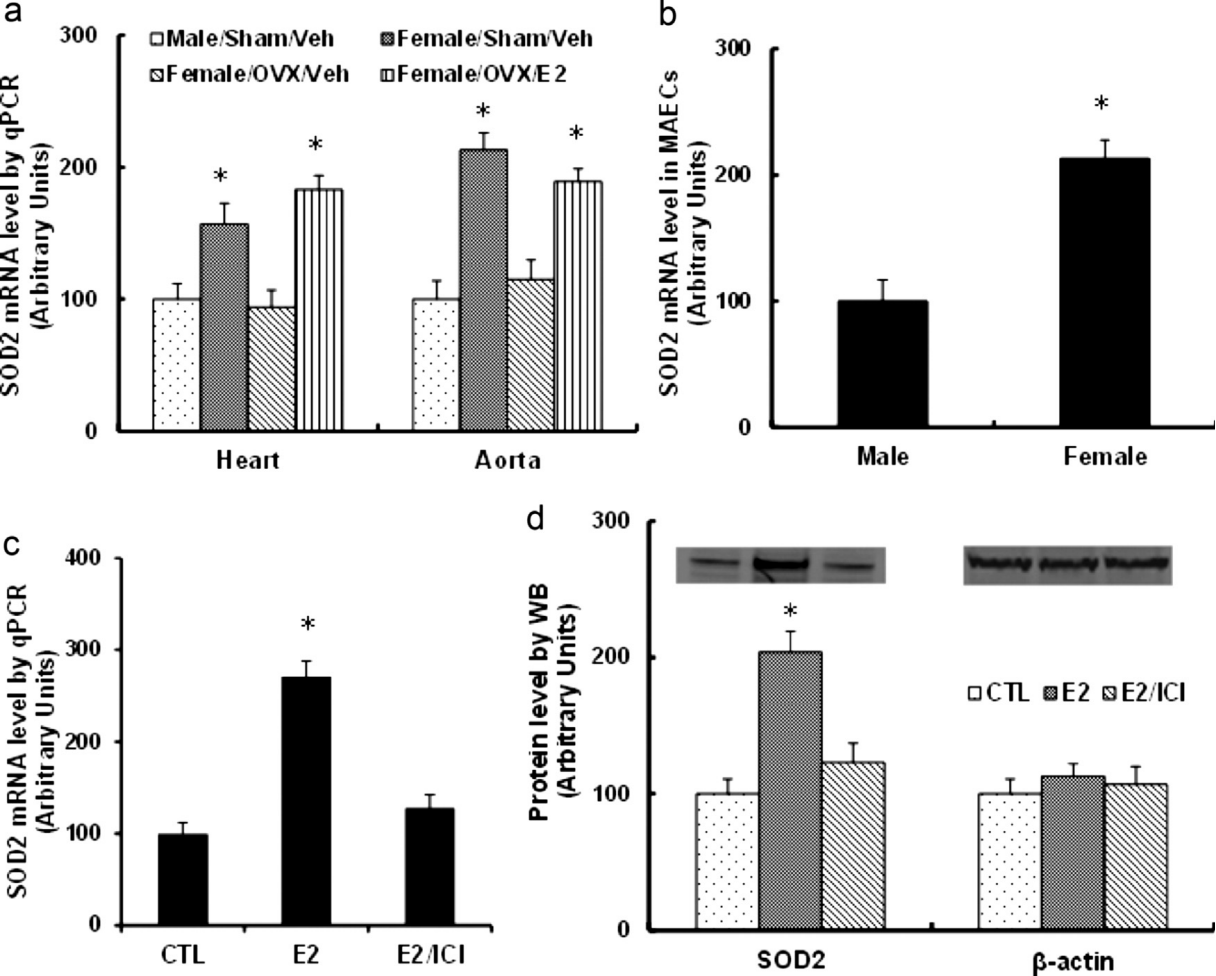
The expression of SOD2 is regulated by E2 and ER. (a, b) 6-week-old age-matched male and female C57BL/6J mice received either Sham or ovariectomy (OVX) surgery in the presence of either vehicle (Veh) or E2 for another 2 weeks, then sacrificed, the mRNA expression was analyzed. (a) The tissues of heart and aorta were isolated for SOD2 mRNA analysis. (b) The mouse aorta endothelial cells (MAECs) were isolated using laser capture microdissection for SOD2 mRNA analysis. ^⁎^, *P*<0.05, vs male group. (c, d) The primary HAECs were treated by either ethanol control (CTL), 100 nM E2 or E2 plus 10 µM ICI 182,780 (ICI) for 2 days, the cells were harvested for gene expression. (c) mRNA by qPCR. (d) Representative bands and quantitative results for SOD2 protein by Western blotting. ^⁎^, *P*<0.05, vs CTL group. *n*=4, data are expressed as mean±SEM.

We first measured the mRNA expression of SOD2 in the cardiovascular system of mouse, including the heart and aorta, and found that the female mice have higher SOD2 mRNA levels than male mice, and ovariectomized (OVX) female mice's expression levels were similar to those of male mice, and the expression was restored to wild type females when OVX female was treated by E2 (see Fig. 1a). We also measured the gene expression from mouse aorta endothelial cells isolated by Laser Capture Microdissection (LCM), it showed that female mice have around 2 times higher expression than male mice (Fig. 1b).

### E2-induced SOD2 expression is restored by ER inhibitor in HAECs

We next measured E2-induced gene expression in human aorta endothelial cells (HAECs). In Fig. 1c, the mRNA expression of SOD2 increased around 2.6-fold under the E2 treatment, while this effect was completely blocked by the treatment of estrogen receptor (ER) inhibitor-ICI 182,780, indicating that E2 induces the gene expression through ER activation. We also measured the protein expression of SOD2 (see Fig. 1c), consistent with the changes of mRNA level, E2 treatment increased SOD2 protein expression, while ICI 182,780 treatment blocked the effect of E2.

### E2-induced SOD2 transcriptional-response element is located at the area of − 300 to −100 with multiple GC box binding sites on the SOD2 promoter

**Fig. 2:**
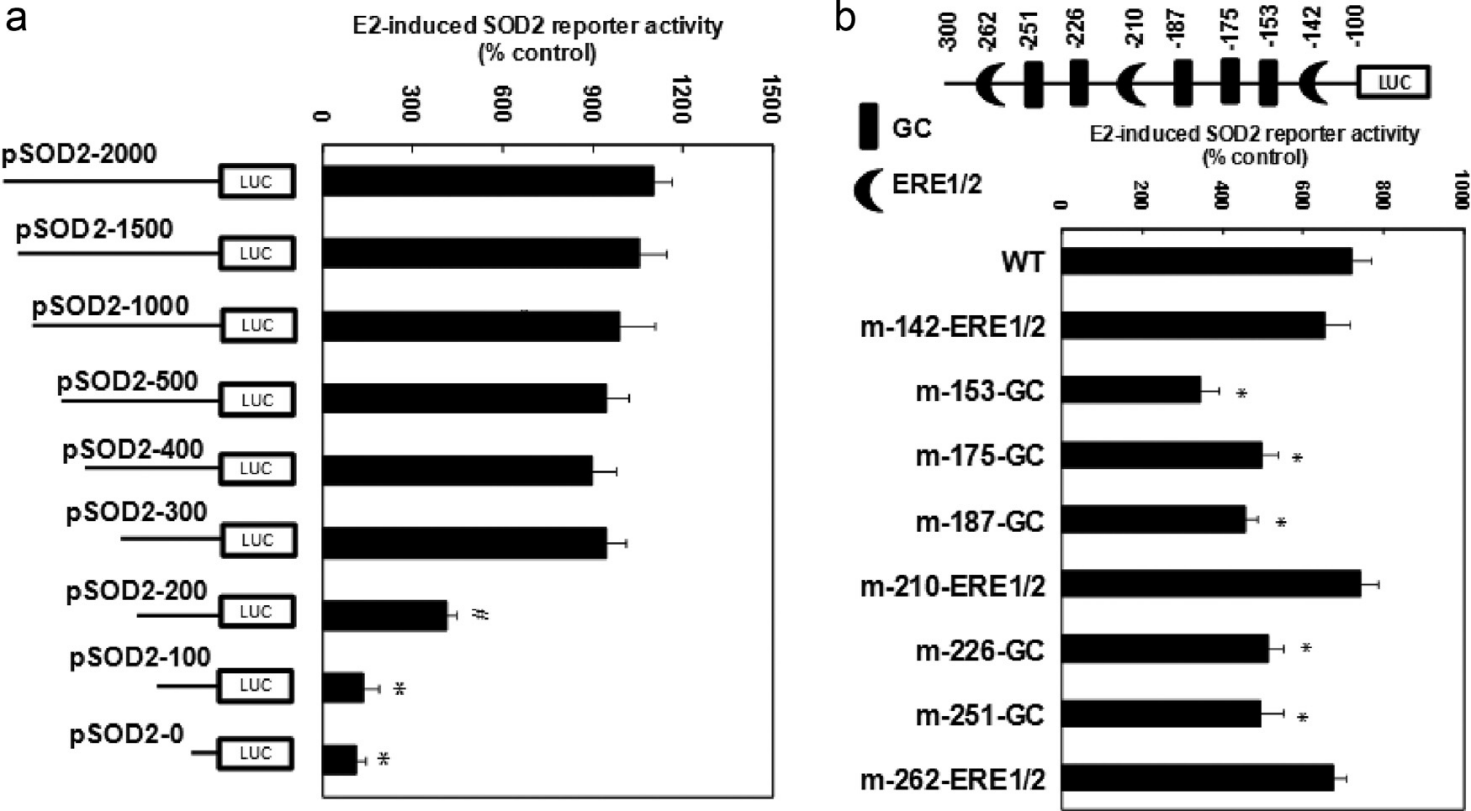
E2-induced SOD2 transcriptional-response element is located at the area of −300 to −100 with multiple GC box binding sites on the SOD2 promoter. (a) The conditional immortalized HAECs were transiently transfected by either SOD2 full length or deletion reporter plasmids, then treated by either control (CTL) or 100 nM E2 for 2 days, and the reporter activities were measured. ^⁎^, *P*<0.05, vs pSOD2-2000 group. (b) The cells were transfected by either wild type (WT), ERE1/2 or GC-box. mutation plasmids on the SOD2 promoter, and treated by CTL or E2, and the reporter activities were measured. ^⁎^, *P*<0.05, vs WT group. *n*=4, data are expressed as mean±SEM.

The mechanism of E2-induced SOD2 expression was further investigated. To localize the regulatory elements required for transcriptional expression of SOD2 gene by E2 treatment, the progressive 5′ promoter deletion constructs, including −2000, −1500, −1000, −500, −400, −300, −200, −100, 0 were generated (numbered according to Ensembl Transcript ID: ENST00000337404). In Fig. 2a, the E2-induced SOD2 transcriptional-response element is located in the area of − 300 to −100 on the SOD2 promoter. Transcription factor databases (TESS) revealed several potential binding motifs in this area, including 5 GC boxes (Sp1 binding site) and 3 ERE1/2 binding sites as shown in Fig. 2b (upper panel). The possible involvement of these motifs on the E2-induced SOD2 transcriptional expression was explored using a series of single mutants in luciferase constructs at the area of − 300 to −100. As shown in Fig. 2b down panel, all of the 5 GC-box single mutants significantly decreased the E2-induced SOD2 activation, whereas none of the ERE1/2 motif showed any effect. This indicates that the multiple GC box binding motif located at − 300 to −100 area is required for E2 responsiveness of the SOD2 promoter.

### E2-induced SOD2 expression is due to the E2-induced ER activation and the subsequent increased binding of ERα/Sp1 and decreased binding of ERβ/Sp3 to multiple GC box located at − 300 to −100 on the SOD2 promoter

**Fig. 3:**
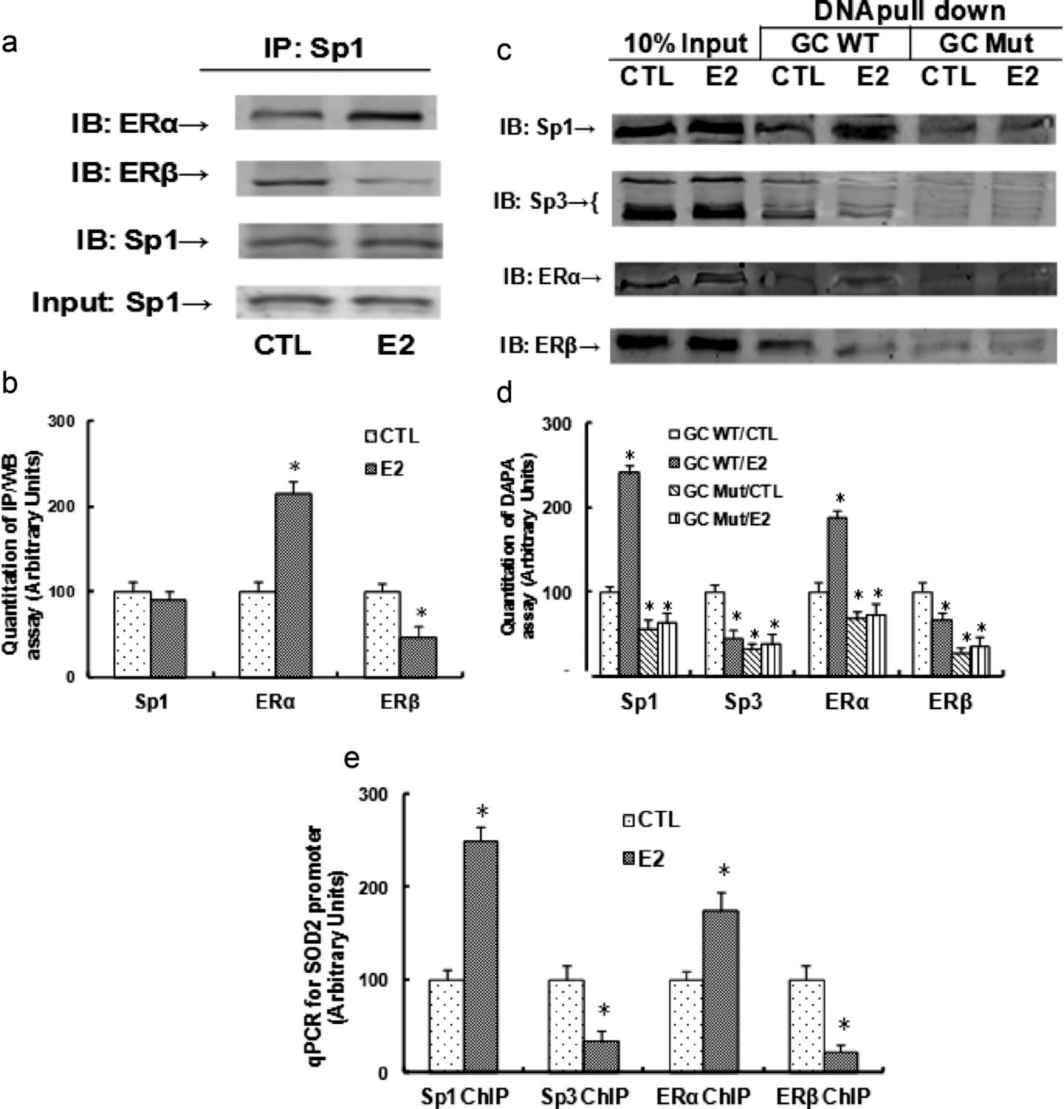
E2/ER activation induces increased binding of ERα/Sp1 and decreased binding of ERβ/Sp3 to multiple GC box on the SOD2 promoter. (a) The nuclear extracts from the above treated cells were immunoprecipitated (IP) by Sp1, then immunoblotted by Sp1, ERα or ERβ respectively. (b) The quantitation results for a. (c) The biotin-labeled oligonucleotides (fragment of −270 to −138 on the SOD2 promoter) were used for DNA pull-down assay in the nuclear extracts from treated cells, and the pull down proteins were blotted by Sp1, Sp3, ERα and ERβ, respectively. (d) The quantitation results for c. (e) The primary HAECs were treated by either CTL or E2 for 2 days, the cells were then harvested for ChIP analysis using the antibodies of ERα, ERβ, Sp1 and Sp3, respectively, and the SOD2 promoter in the range of −100 to −262 was amplified by qPCR. ^⁎^, *P*<0.05, vs CTL group. *n*=4, data are expressed as mean±SEM.

We first did the immunoprecipitation (IP) using Sp1 antibody from treated cellular nuclear extracts, then immunoblotted (IB) by ERα and ERβ. We found that E2 treatment significantly increased the association of Sp1 with ERα, but decreased the association of Sp1 with ERβ (see Fig. 3a, b). On the other hand, we obtained negative results from IP/IB for Sp3/ERβ, indicating that either Sp3 does not directly bind with ERβ, or the interaction of Sp3 and ERβ is too weak to be detected. We then investigated the binding ability of those transcription factors on the above multiple GC-box of SOD2 promoter under the E2 treatment. The biotin-labeled DNA oligonucleotides (fragment of − 270 to −138 on the SOD2 promoter) were used for DNA pull down assay (DABA assay) as shown in Fig. 3c, d. The results showed that E2 treatment significantly increased the binding affinity of ERα and Sp1, and decreased the affinity of ERβ and Sp3 on the indicated wild type fragment (GC WT), while the fragment with GC box mutants (GC Mut, mutation points of −153, −175, −187, −266, −251) decreased the binding ability of Sp1, Sp3, ERα and ERβ, also, it largely diminished the effect of E2. Finally, we did the chromatin immunoprecipitation (ChIP) analysis using Sp1, Sp3, ERα and ERβ antibodies respectively on the SOD2 promoter in the area of − 300 to −100. Results showed that E2 treatment decreased the association of Sp3 and ERβ, but increased the association of Sp1 and ERα on the SOD2 promoter (see Fig. 3e).

### ERβ/Sp1/Sp3 play a critical role in maintaining the basal level of SOD2 expression, while ERα/Sp1 responses E2-induced SOD2 up-regulation

**Fig. 4:**
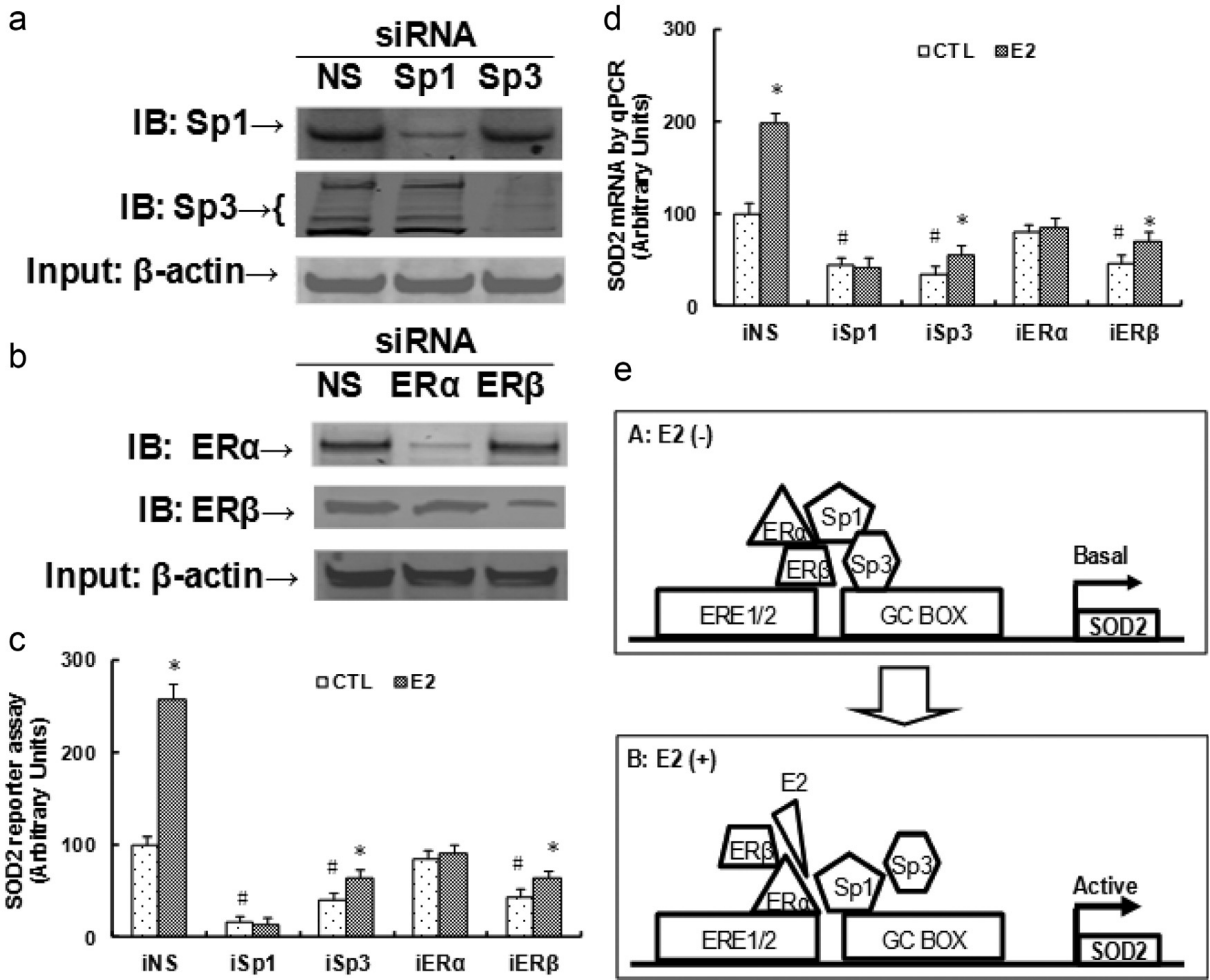
ERβ/Sp1/Sp3 play a critical role in maintaining basal level of SOD2 expression, while ERα/Sp1 responses E2-induced SOD2 up-regulation. Conditionally immortalized HAECs were transfected by siRNA for NS (non-sense) control, Sp1 or Sp3, ERα or ERβ, plus luciferase reporter plasmids, then the cells were treated by either ethanol control (CTL) or E2 for 2 days, and the cells were harvested for the measurement. (a) Western blots for Sp1 and Sp3 after siRNA treatment. (b) Western blots for ERα and ERβ after siRNA treatment. (c, d) The above siRNA treated cells were harvested for SOD2 reporter activity assay (c) and mRNA analysis (d). ^⁎^, *P*<0.05, vs CTL group; ^#^, *P*<0.05, vs CTL in iNS group. (e) Schematic model for E2-induced SOD2 up-regulation. *n*=4, data are expressed as mean±SEM.

We then evaluated the contribution role of Sp1, Sp3, ERα and ERβ on the SOD2 expression using siRNA techniques. The above mentioned transcription factors were knocked down in conditionally immortalized HAECs, and the protein levels were confirmed by western blotting (see Fig. 4a, b). We then measured the SOD2 luciferase reporter activity in those knockdown cells as shown in Fig. 4c. We found that knockdown of ERβ and Sp3 significantly decreased SOD2 basal reporter activity, but the cells still respond to E2-induced activation, even though the basal level is much lower than wild type cells. Knockdown of ERα did not significantly decrease the basal reporter activity, but completely abolished E2-induced SOD2 reporter activation. Sp1 knockdown largely decreased the basal SOD2 reporter activity, also, it diminished the E2-mediated SOD2 reporter activation. We then measured the SOD2 mRNA expression from those knockdown cells, the results were similar with SOD2 reporter activity (see Fig. 4d). This suggests that Sp3 and ERβ play important role to maintain basal SOD2 expression level, ERα responses E2-induced SOD2 up-regulation, while Sp1 not only responses basal expression, but also responses E2-induced SOD2 up-regulation. Finally, in Fig. 4e, we established the schematic model for E2-induced SOD2 expression. In the absence of E2, the complex of ERβ and Sp3 have a higher binding ability to GC-box than the complex of ERα and Sp1, this keeps the basal level of SOD2 expression. While in the presence of E2, activated ERα has a higher association with Sp1, this complex competitively binds to the GC-box with ERβ/Sp3 and up-regulates the SOD2 expression.

### E2-induced SOD2 up-regulation minimizes the ROS generation in HAECs

**Fig. 5:**
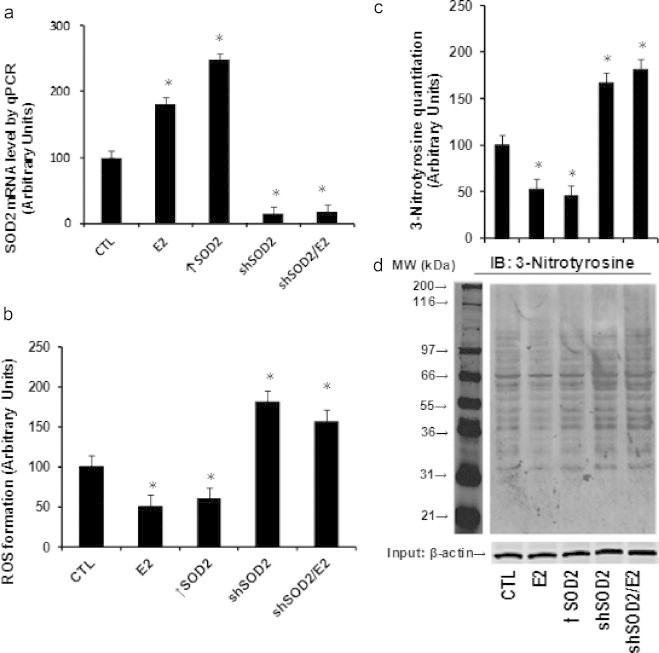
E2/ER-mediated SOD2 up-regulation minimizes the ROS generation in HAECs. Conditionally immortalized HAECs were treated by control (CTL), E2, or infected by lentivirus for SOD2 expression (↑SOD2) or knockdown (shSOD2) in the presence or absence of E2 for 2 days, the cells were harvested for the analysis. (a) mRNA analysis of SOD2 by qPCR. (b) ROS generation. (c) Quantitation results for 3-nitrotyrosine accumulation from western blotting. (d) Representative lanes for the 3-nitrotyrosine staining. ^⁎^, *P*<0.05, vs CTL group. *n*=4, data are expressed as mean±SEM.

The molecular consequence of E2-induced SOD2 up-regulation was investigated. Conditionally immortalized HAECs were either treated by control (CTL), E2, or infected by either SOD2 expression (↑SOD2) or knockdown lentivirus (shSOD2) with the presence or absence of E2 for 2 days, and the cells were used for further analysis. In Fig. 5a, the SOD2 mRNA level was measured, we found that E2 treatment or SOD2 lentivirus infection increased SOD2 expression significantly, while shSOD2 lentivirus largely decreased SOD2 expression regardless of the E2 treatment. Next, we measured the in vitro ROS generation as shown in Fig. 5b, the ROS generation was decreased around 50% with the E2 treatment, and the SOD2 lentivirus infection (↑SOD2) decreased the ROS generation by ~53% compared to control group, which mimicked the effect of E2. On the other hand, the SOD2 knockdown (shSOD2) increased ROS by ~1.7-fold regardless of the E2 presence, indicating that the SOD2 expression plays a key role in the level of ROS generation. Finally, we measured the 3-nitrotyrosine (3-NT) accumulation through western blots. In Fig. 5c, d, the results showed that E2 treatment significantly decreased 3-NT accumulation, and the SOD2 infection mimicked this effect, while SOD2 knockdown (shSOD2) treatment increased 3-NT accumulation by ~1.6-fold, even in the presence of E2. These data suggest that E2-induced SOD2 up-regulation minimizes the ROS generation in HAECs.

### Tie2-driven SOD2 lentivirus infection on vascular wall specifically and efficiently modulates the SOD2 activity in endothelial cells

**Fig. 6:**
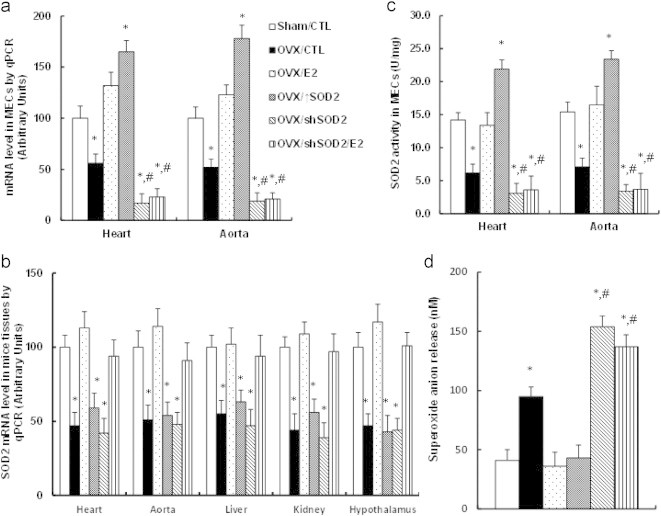
Endothelium-specific delivery of SOD2 expression on vascular wall mimics E2-induced minimized ROS generation, and the SOD2 knockdown totally diminishes E2-induced effect. (a) Treated mice were sacrificed, the mouse endothelial cells (MECs) were isolated by laser capture microdissection, and the SOD2 mRNA level was measured, *n*=5. (b) The treated mice were sacrificed and the related tissues, including heart, aorta, liver, kidney and hypothalamus were isolated for the analysis of SOD2 mRNA, *n*=4. (c) The mouse endothelial cells (MECs) from the heart and aorta were isolated and cultured for in vitro analysis of SOD2 activity, *n*=4. (d) The whole aortas from treated mice were isolated for in vivo superoxide release analysis, *n*=6. ^⁎^, *P*<0.05, vs sham/CTL group; ^#^, *P* < 0.05, vs OVX/CTL group. Data are expressed as mean±SEM.

In order to further confirm our findings that E2-induced SOD2 up-regulation has a vasculoprotective effect in endothelial cells, the HFD treated mice were infected by SOD2 expression (↑SOD2) or knockdown (shSOD2) lentivirus through tail vein injection, and the SOD2 expression was measured. In Fig. 6a, the mouse endothelial cells (MECs) from the heart and aorta were isolated using Laser Capture Microdissection (LCM) for the mRNA analysis, the results showed that the removal of E2 through OVX surgery significantly decreased the expression of SOD2 by ~2-fold compared to the Sham/CTL mice, the E2 treatment on those OVX mice restored the SOD2 expression, while the SOD2 lentivirus infection of those OVX mice significantly increased SOD2 expression by ~1.6- and ~1.8-fold respectively compared to Sham/CTL mice. On the other hand, the shRNA SOD2 knockdown (shSOD2) decreased the basal SOD2 expression for more than 4-fold compared to the Sham/CTL mice, and the E2 treatment had no effect on SOD2 expression as long as SOD2 was knocked down. We also measured the SOD2 mRNA expression from other tissues, including heart, aorta, live, kidney and hypothalamus as shown in Fig. 6b, we found that OVX surgery decreased SOD2 expression by ~50% in those tissues, and the E2 treatment (from OVX/E2 group) significantly increased the SOD2 expression in all the tissues compared to OVX/CTL group, neither SOD2 expression (↑SOD2) nor SOD2 knockdown (shSOD2) treatment alone had any effect on the SOD2 expression, while the treatment of SOD2 knockdown plus E2 treatment (OVX/shSOD2/E2) significantly increased the SOD2 expression. It is interesting that we observed significant changes of SOD2 expression in MECs isolated from both heart and aorta with the lentivirus infection, while there were no significant changes from the whole heart, aorta and other tissues, this may be explained by the fact that the percentage of endothelial cells from the whole tissue is very low, and the expression changes from such a small percentage of endothelial cells cannot be distinguished from the whole tissue. Our data suggest that Tie2-driven SOD2 lentivirus infection is efficient and specific. We then investigated the SOD2 activity from cultured mice endothelial cells (MECs) that were isolated from the heart and aorta. In Fig. 6c, OVX surgery significantly decreased SOD2 activity by ~2-fold, the E2 treatment normalized this effect, and the SOD2 infection (OVX/E2) largely increased SOD2 activity by ~1.5-fold compared to control group (Sham/CTL), while the SOD2 knockdown (shSOD2) significantly decreased SOD2 expression by ~4-fold regardless of the E2 presence, indicating that the infection of SOD2 lentivirus is efficient in modulating the SOD2 activity.

### SOD2 lentivirus infection mimics the E2 treatment with minimized superoxide anion release in the aorta

We investigated the effect of SOD2 lentivirus infection on the ROS generation from those aortas. In Fig. 6d, the in vivo superoxide anion (O_2_^•−^) release from the whole aorta was measured. The OVX surgery increased the ROS generation by more than 2-fold compared to Sham/CTL mice, and the E2 treatment or SOD2 lentivirus infection (↑SOD2) restored OVX-induced ROS generation. On the other hand, the SOD2 knockdown in OVX-treated mice (OVX/shSOD2) also significantly increased superoxide release despite the E2 treatment compared to OVX treatment (OVX/CTL). Our data indicate that the E2 modulates the ROS generation through the regulation of SOD2 expression.

### Tie2-driven lentivirus Infection of SOD2 on the vascular wall ameliorates the E2 deficiency-induced mitochondrial dysfunction and vascular damage, while SOD2 knockdown partly diminishes the E2 effect

**Fig. 7:**
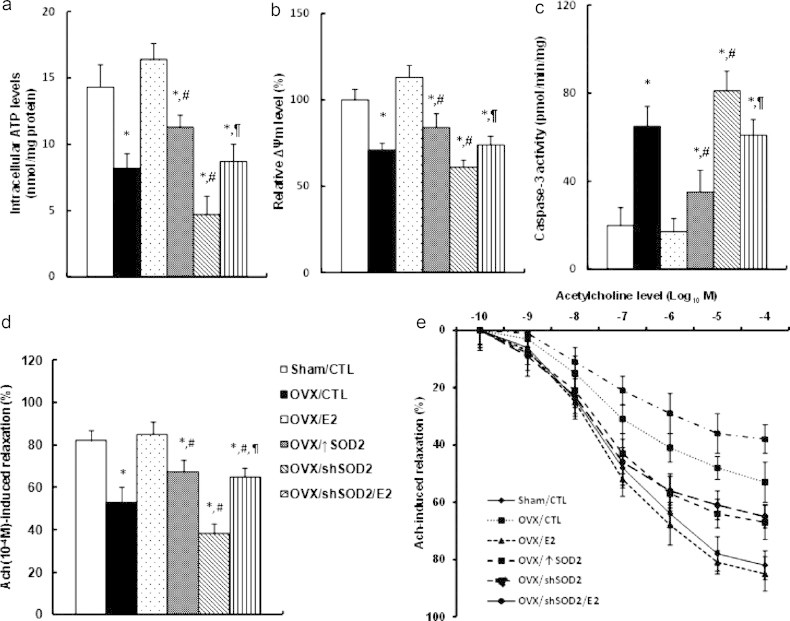
Endothelium-specific delivery of SOD2 expression on vascular wall ameliorates the mitochondrial dysfunction and vascular damage, while SOD2 knockdown partly diminishes E2-induced effect. (a–c) The treated mice were sacrificed, the mouse endothelial cells (MECs) from the heart or aorta were isolated and cultured for in vitro analysis. (a) Intracellular ATP levels; (b) relative mitochondrial membrane potential (Δ*Ψ*_m_); (c) caspase-3 activity. *n*=5. (e, f) Treated mice were sacrificed, the aortas were dissected for the vessel tension analysis. The rings were preconstricted with phenylephrine, and the acetylcholine (Ach, 10^−10^–10^−4^ M) was injected at the plateau of the phenylephrine-induced contraction. (e) The 10^−4^ M Ach-induced aorta ring relaxation. (f) The ach-induced aorta ring relaxation curves, *n*=8–10. ^⁎^, *P*<0.05, vs Sham/CTL group; ^#^, *P*<0.05, vs OVX/CTL group; ^¶^, *P*<0.05, vs OVX/shSOD2 group. Data are expressed as mean±SEM.

In order to evaluate the effect of E2 and the SOD2 manipulation on the vascular wall, the MECs were isolated and cultured for the in vitro analysis. We measured several markers to evaluate the mitochondrial function, including the intracellular ATP level, the mitochondria membrane potential (MMP, Δ*Ψ*_m_) and the caspase-3 activity as shown in Fig. 7a, b and c, respectively. The results showed that OVX surgery significantly decreased the intracellular ATP level, the mitochondrial membrane potential, and increased the caspase-3 activity compared to control group (Sham/CTL), this effect was completely restored by the E2 treatment, while the SOD2 infection (↑SOD2) can only partly restore this effect. Furthermore, the OVX-induced mitochondria dysfunction was worsened by the shSOD2 knockdown, while the E2 treatment on OVX/SOD2 knockdown mice (OVX/shSOD2/E2) significantly ameliorated the effect, but again, cannot completely restore to normal (Sham/CTL), indicating that E2-induced SOD2 plays important vasculoprotective role, while some other factors may be also involved in this process. Finally, the vessel tension of the vascular ring was measured to evaluate the effect of the E2 treatment and the SOD2 manipulation on the vascular wall. As shown in Fig. 7d, e the acetylcholine-induced relaxation was significantly decreased by OVX surgery compared to control (CTL) group, and this effect was restored by the E2 treatment, but partly restored by the SOD2 infection. On the other hand, the OVX-induced decreased relaxation was worsened by the SOD2 knockdown, and again, the E2 treatment can only partly restore this effect. This implies that some other kinds of E2-induced factors may be also involved in E2-deficiency induced vascular damage. Our results prove that the E2-induced up-regulation of SOD2 in endothelial cells has a vasculoprotective effect.

## Discussion

Our study demonstrates that estrogen/estrogen receptor up-regulates the SOD2 expression in endothelial cells with minimized ROS generation and ameliorated cardiovascular damage. To our knowledge, this is the first time we have shown the potential molecular mechanism for estrogen-mediated vasculoprotective effect through up-regulation of specific gene on vascular wall.

### Dominant role of endothelial cells in E2-induced vasculoprotective action

Several cell populations in cardiovascular system, including endothelial cells, vascular smooth muscle cells (VSMC) and inflammatory immune cells (monocytes–macrophages, lymphocytes) play crucial role in the pathophysiology of atherosclerosis [4,34–36], while recent study shows that the endothelial cells play a dominant role in E2-induced vasculoprotective action [4,34]. In this study, we chose HAECs as in vitro cell model to investigate the mechanism of E2-induced gene expression. Also, in the in vivo mice experiments, we used endothelium-specific Tie2-promoter to drive the SOD2 expression in a lentivirus vector, this vector will only express the target genes in endothelial cells. On realizing that the Tie2-promoter driven expression may have some leaking effects, especially on hematopoietic lineages, we measured the gene expression from other tissues. The results showed that the SOD2 expression was only increased in endothelial cells from the heart and aorta, showing no increase in liver, kidney and hypothalamus (see Fig. 6a, b), indicating that Tie2 driven lentivirus infection is sufficient. This can be explained by the recent finding that endothelial ERα plays a key role on the E2-induced atheroprotective effect, while the hematopoietic ERα is dispensable [4].

### Role of ERα/β and Sp1/Sp3 in E2-induced SOD2 expression

E2 has been reported to have an antioxidant effect, it protects against the oxidation of LDL, modulates the antioxidant gene expression etc. [37,38], while the exact mechanism still needs to be fully understood. Here, we have identified a detailed molecular mechanism for E2-induced SOD2 expression in endothelial cells, up-regulated SOD2 expression minimizes ROS formation and 3-NT accumulation, and subsequently ameliorate E2 deficiency-induced vascular dysfunction. We have shown that the SOD2 expression is mediated through E2-induced ERα/ERβ activation and subsequent tethered Sp1/Sp3 binding to the GC box [39], where ERα responses SOD2 activation, and ERβ responses SOD2 basal expression in vascular endothelial cells, knockdown of ERα does not significantly decrease the SOD2 basal expression. This indicates that both ERα and ERβ play important role in E2-induced vasculoprotective action [40,41]. In fact, many studies have shown that ERα plays a dominant role in E2-induced vasculoprotective effect [4,5,42], while using ERα and/or ERβ null mice, both ERs have shown to be necessary and sufficient for E2-mediated vascular protection [43], and recently, ERβ has indicated that it plays a crucial role in preventing early-stage atherosclerosis in ERα deficient mice [13]. The above contradictory observations can be explained well by our findings.

### Vasculoprotective role of SOD2 and E2 in high-fat diet mice

Recent evidence indicates that reactive oxygen species (ROS) produced in mitochondria contributes to the pathogenesis of cardiovascular diseases [44,45], and the ROS plays many important roles in triggering vascular damage, including oxidized LDL, ROS induced DNA damage, mitochondrial dysfunction, impaired enzyme activity etc. [23,44,46]. In our study, we show that SOD2 expression in OVX mice can only partly restore E2-deficiency induced mitochondrial dysfunction and vascular damage, indicating that some other factors may also be involved in this process. In fact, the E2 has been reported to play many important vasculoprotective roles except its antioxidant effect, which include an anti-inflammatory effect, regulation of feeding, food intake and energy balance to prevent obesity [7,47], induction of eNOS expression [48], preventing the cell death and mitochondrial dysfunction [49], and preventing the fat accumulation in high-fat diet mice etc. [7], all of them favor cardiovascular function [50]. In this scenario, it is not surprising that E2-induced SOD2 expression can only ameliorate E2-deficiency induced vascular damage, instead of completely restoring that effect.

Clinical studies show that high-fat diet (HFD) impairs endothelial function in humans [51], and HFD-induced obese mice have been used to replicate the features of human obesity [52] with many cardiovascular dysfunctions, including inflammation, hypertrophy, fibrosis, apoptosis, and endothelial dysfunction etc. [53–55]. In our study, we used HFD mice as animal model to investigate the effect of E2 and SOD2 expression on the vascular wall, our results show the significant vascular damage reflected by decreased vessel tension in OVX/SOD2 knockdown mice (OVX/shSOD2) compared to the control group (Sham/CTL) as shown in Fig. 6d, e, while we did not find any significant changes of hypertension and atherosclerotic lesions [56] in our treated mice, this is consistent with the previous findings that many obese patients develop hypertension, while 40–60% of them do not [57].

Altogether, our findings show that E2-induced vasculoprotective mechanism is partly due to E2-induced SOD2 expression in endothelial cells. Gene therapy through infection of SOD2 on vascular wall may ameliorate E2 deficiency-induced cardiovascular damage in postmenopausal women.

## Figures and Tables

**Table 1:** Details and conditions for the mice treatment.

Animal group	Sham/CTL	OVX/CTL	OVX/E2	OVX/↑SOD2	OVX/shSOD2	OVX/shSOD2/E2
*n*	13	15	14	17	13	16
Sham/OVX surgery	Sham	OVX	OVX	OVX	OVX	OVX
Vehicle/E2 pellet	Vehicle	Vehicle	E2	Vehicle	Vehicle	E2
Lentivirus injection	Tie2-empty	Tie2-empty	Tie2-empty	Tie2-↑SOD2	Tie2-ShSOD2	Tie2-shSOD2
Plasma E2 (pg/ml)	47±9	14±3	105±13	13±4	15±5	101±15
Body weight (g)	36.2±1.2	42.6±1.1	37.6±1.5	40.7±1.4	41.8±1.3	38.9±1.0
Total cholesterol (mg/dl)	146.1±14.3	279.4±13.4	139.7±12.1	265.3±14.9	281.4±15.4	144.3±15.3
Triglyceride (mg/dl)	128.4±8.7	208.6±10.4	129.7±7.9	205.1±9.7	211.7±14.6	133.4±9.3
